# Weaving Together: Enhancing KAER to Optimize Conversations Between Providers & American Indian Family Caregivers

**DOI:** 10.1093/geroni/igaf122.1928

**Published:** 2025-12-31

**Authors:** Maria Donohoe, Anne Helene Skinstad, Sato Ashida, Lena Thompson

**Affiliations:** University of Iowa, Iowa City, Iowa, United States; University of Iowa College of Public Health, Iowa City, Iowa, United States; University of Iowa, Iowa City, Iowa, United States; University of Alaska Fairbanks, Fairbanks, Alaska, United States

## Abstract

American Indian and Alaska Native (AI/AN) populations experience higher rates of dementia than other racial/ethnic groups in the U.S. Toolkits such as Kickstart Assess Evaluate, and Refer (KAER) can help primary care providers start conversations with people experiencing cognitive decline and their families, facilitating dementia diagnosis and care. Yet, preliminary conversations with members from a midwestern tribe highlighted cultural incongruence in KAER contents. To conduct this SAMHSA-funded project to enhance the KAER model, the research team engaged two members from a Midwest Tribe who had cared for someone with dementia, one provider from the midwestern tribe, and a Native American Elder justice specialist. Participants completed cognitive interviews, in-depth interviews, and document reviews to enhance KAER. Along with enhancing KAER, the research team responded to participant feedback that a toolkit was needed for AI/AN caregivers to begin conversations with providers about dementia. The Communication Assess Review Essential referrals and Self-care (CARES) toolkit was developed for AI/AN caregivers. The CARES and enhanced KAER included mental and physical health topics, language modifications, Tribe-specific community resources, and a section on self-care. CARES and enhanced KAER also included case studies sharing experiences of caregivers from the midwestern tribe to provide support and examples to AI/AN caregivers and their providers. Enhancements to existing toolkits such as KAER can support AI/AN caregivers and reduce ADRD disparities. Including community members in early conversations and assessments can support community-engaged intervention enhancement by informing culturally relevant programs and tools. Future work to test the adapted toolkit is needed.

